# Contactless and continuous monitoring of respiratory rate in a hospital ward: a clinical validation study

**DOI:** 10.3389/fphys.2024.1502413

**Published:** 2024-11-27

**Authors:** Ståle Toften, Jonas T. Kjellstadli, Jørn Kværness, Line Pedersen, Lars E. Laugsand, Ole K. F. Thu

**Affiliations:** ^1^ Department of Research and Data Science, Vitalthings AS, Trondheim, Norway; ^2^ God Klinisk Forskning, Oppdal, Norway; ^3^ Department for Pain and Complex Disorders, St. Olavs University Hospital, Trondheim, Norway; ^4^ Department of Circulation and Medical imaging, Faculty of Medicine and Health Sciences, Norwegian University of Science and Technology (NTNU), Trondheim, Norway; ^5^ Emergency Department, St. Olavs University Hospital, Trondheim, Norway; ^6^ Vitalthings AS, Trondheim, Norway; ^7^ Department of Anesthesia and Intensive Care Medicine, St. Olavs University Hospital, Trondheim, Norway

**Keywords:** contactless monitoring, continuous monitoring, monitoring, respiratory rate, hospital, clinical deterioration, validation, Vitalthings Guardian M10

## Abstract

**Introduction:**

Continuous monitoring of respiratory rate in hospital wards can provide early detection of clinical deterioration, thereby reducing mortality, reducing transfers to intensive care units, and reducing the hospital length of stay. Despite the advantages of continuous monitoring, manually counting every 1–12 h remains the standard of care in most hospital wards. The objective of this study was to validate continuous respiratory rate measurements from a radar-based contactless patient monitor [Vitalthings Guardian M10 (Vitalthings AS, Norway)] in a hospital ward.

**Methods:**

An observational study (clinicaltrials.gov: NCT06083272) was conducted at the emergency ward of a university hospital. Adult patients were monitored during rest with Vitalthings Guardian M10 in both a stationary and mobile configuration simultaneously with a reference device [Nox T3s (Nox Medical, Alpharetta, GA, United States)]. The agreement was assessed using Bland-Altman 95% limits of agreement. The sensitivity and specificity of clinical alarms were evaluated using a Clarke Error grid modified for continuous monitoring of respiratory rate. Clinical aspects were further evaluated in terms of trend analysis and examination of gaps between valid measurements.

**Results:**

32 patients were monitored for a median duration of 42 min [IQR (range) 35–46 (30–59 min)]. The bias was 0.1 and 0.0 breaths min^−1^ and the 95% limits of agreement ranged from −1.1 to 1.2 and −1.1 to 1.1 breaths min^−1^ for the stationary and mobile configuration, respectively. The concordances for trends were 96%. No clinical alarms were missed, and no false alarms or technical alarms were generated. No interval without a valid measurement was longer than 5 min.

**Conclusion:**

Vitalthings Guardian M10 measured respiratory rate accurately and continuously in resting patients in a hospital ward.

## 1 Introduction

To reduce mortality, transfers to intensive care units (ICUs), activations of rapid response teams, and hospital length of stay, vital signs should be monitored continuously (Sun et al., 2020; Downey et al., 2018). Changes in respiratory rate have been emphasised as particularly important for early detection of clinical deterioration (Churpek et al., 2016; Akel et al., 2021; Mochizuki et al., 2017). Timely detection of conditions like opioid-induced respiratory depression, primarily caused by reduced respiratory rate leading to hypoventilation (Algera et al., 2019), could reduce complications and save hospital cost. A large multi-center study found that 46% of all patients receiving opioids in general wards experienced at least one episode of respiratory depression, and their hospital stay was, on average, 3 days longer compared to those without an episode (Khanna et al., 2020). Another study revealed that a substantial number of severe opioid-induced respiratory depressions occurred within 15 min of the last nursing check (Lee et al., 2015), highlighting the need for continuous monitoring. Continuous respiratory rate monitoring has been shown to halve the incidents of opioid-induced respiratory depression in patients using patient-controlled analgesia (Stites et al., 2021). Respiratory rate also emerges as the most reliable vital sign in predicting in-hospital cardiac arrests (Churpek et al., 2012), which are estimated to occur 290,000 times per year in the United States alone (Andersen et al., 2019). Notably, continuous monitoring has been shown to double survival rates (Perman et al., 2016). Furthermore, a study examining preventable deaths in hospitals revealed that a substantial portion can be attributed to inadequate clinical monitoring (Lee et al., 2015). Despite the advantages of continuous monitoring of respiratory rate, manual spot checks performed every 1–12 h remain the standard of care in most hospital wards, despite their limitations in capturing real-time changes (Physicians, 2021). Moreover, these spot checks are time-consuming and inaccurate (Jack et al., 2017; Palmer et al., 2023; Lim et al., 2002; Kallioinen et al., 2021).

Medical devices that monitor respiratory rate today have several limitations. Impedance pneumography is commonly used in ICUs, but its tendency to trigger false alarms contributes to alarm fatigue—a significant concern for patient safety (Bawua et al., 2021; Drew et al., 2014; Albanowski et al., 2023; Sendelbach and Funk, 2013; Ruskin and Hueske-Kraus, 2015). This issue is particularly challenging in settings with fewer nurses per patient, thereby limiting the effectiveness of impedance pneumography outside the ICU. Moreover, the patient’s wired connection to a monitor complicates its usage. While capnography is regarded as the gold standard for measuring respiratory rate in intubated patients, it is often not suitable for non-intubated patients due to frequent sensor malposition and poor patient acceptance (Breteler et al., 2020; Marjanovic et al., 2020). Less intrusive alternatives have been developed such as under-the-mattress sensors, acoustic sensors, and wearable patches, but studies have found their accuracy to be clinically unacceptable for respiratory rate monitoring (Chan et al., 2022). Other non-intrusive alternatives have emerged such as cameras, radars, and optical sensors, but they are not approved for high-risk situations (Chan et al., 2022). Thus, the demand persists for technology that can monitor respiratory rate accurately, reliably, and non-intrusively in hospital wards (Marjanovic et al., 2020; Michard and Kalkman, 2021). Moreover, proper validation of these devices focusing on clinical aspects of continuous monitoring is essential (Saugel et al., 2020).

This study aimed to validate continuous respiratory rate measurements obtained from a radar-based contactless patient monitor in a hospital ward.

## 2 Material and methods

The study was approved by the Norwegian Medical Products Agency (CIV-NO-23-08-043752) and the regional ethical committee (REK KULMU B 617617). Written informed consent was obtained from all participants and the study was conducted in accordance with the principles embodied in the Declaration of Helsinki (Association, 2013). The study was pre-registered at clinicaltrials.gov the 8th of October 2023 (NCT06083272) and adheres to the applicable STROBE guidelines (von Elm et al., 2008).

### 2.1 Study design

The study was an observational confirmatory study conducted during daytime at the emergency ward of a university hospital (St. Olavs Hospital, Trondheim, Norway) from November 15th to 28th, 2023. Respiratory rates of patients resting in bed were measured simultaneously for 30 min to 4 h using a contactless patient monitor (Vitalthings Guardian M10, Vitalthings AS, Norway) and a reference device (Nox T3s, Nox Medical, Alpharetta, GA, United States). Monitoring was paused during doctor visits/examinations. Monitoring was ceased if the patient left the study room for further examination, was transferred from the emergency ward, or had been monitored for 4 h. The study did not interfere with standard care. The study was a part of the clinical investigation required for MDR approval of the Vitalthings Guardian M10.

### 2.2 Study population

To include patients with a diverse range of medical and surgical conditions, recruitment was conducted in the emergency ward. Recruitment was done after the initial triage, either while waiting for an examination or control test, or while waiting to be transferred to a hospital ward after the treatment and examination in the emergency ward were finished. Patients were invited to participate only if an emergency medicine specialist regarded it safe for the patient and the study would not affect the patient’s care.

Inclusion criteria were adult patients admitted to the emergency ward. Exclusion criteria were patients less than 18 years old and subjects not able to provide informed consent. Subjects monitored for less than 30 min were removed from the study to assure a certain number of measurements per person and to enable reasonable gap analysis of continuity.

### 2.3 Vitalthings Guardian M10

Vitalthings Guardian M10 (hereafter Guardian M10) is an MDR class IIb approved contactless patient monitor designed for use in wards at healthcare facilities. It employs an ultrawideband radar to transmit electromagnetic pulses which are reflected by the human body and received by the device. Based on changes in the received signal, movements can be detected. Movements due to respiration are extracted to create and display a respiratory waveform. The respiratory rate is derived from the waveform by counting peaks over 1 minute. This procedure is repeated to update the respiratory rate every second. The device is configured to measure respiratory rate from 2 to 60 breaths min^−1^. The operating frequencies (7.29 GHz centre frequency) enables the device to measure through clothes and bedsheets, while a directive antenna ensures that only the person in front of the device is measured. The radiated effect of the radar is harmless to humans and complies with EU and North American regulations. The device functions equally well in both dark and light rooms, and no calibration is needed.

**FIGURE 1:**
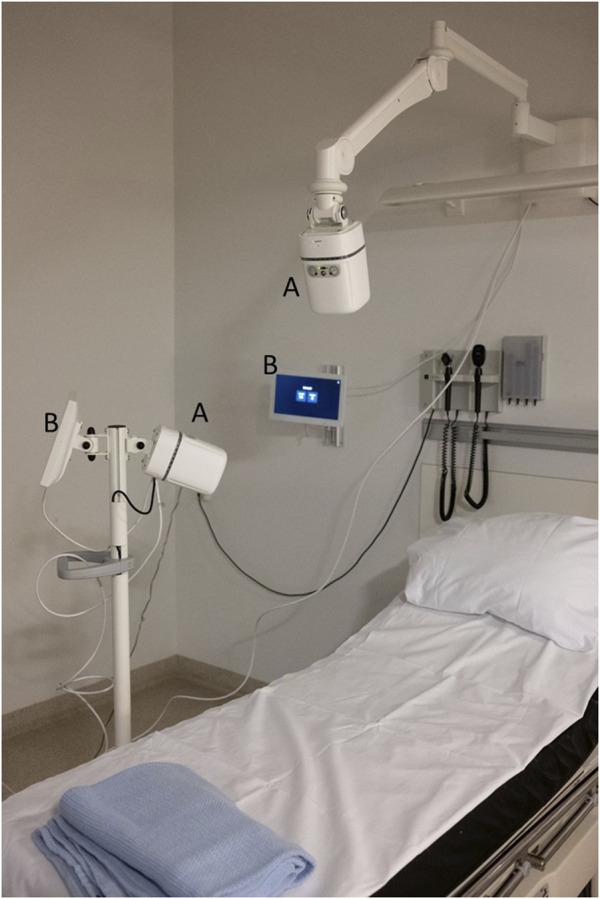
Study setup. The picture shows the Guardian M10 **(A)** with the display tablet **(B)**. One device is stationary wall mounted, whereas the other is mounted on a mobile trolley. Additional cables are connected to a computer to record data (not part of the product).

The Guardian M10 features a touch display and can be connected to centralised monitoring systems. Manufacturer-approved medical devices can be connected to the Guardian M10 to monitor additional vital signs. The device comes in a stationary configuration on an arm and in a mobile configuration on a trolley. The stationary configuration is powered by a cable, while the mobile setup relies on batteries that last at least 14 h. The batteries can be hot-swapped to ensure uninterrupted measurements, allowing for continuous monitoring as long as needed. Both configurations were used in this study in case the sensor position affected the measurements. Devices were positioned so that patients were directly in front of the device (within a 45-degree angle) and operated by a study nurse according to the instructions for use. Figure 1 shows the study setup. In this study, each device was connected to a computer by a cable to record respiratory rates and raw radar data. The raw radar data were used to calculate movement, which was used to synchronise the respiratory rates with the reference values. The respiratory rates were recorded as decimal numbers and were not modified in any way during the study.

### 2.4 Reference

There is no gold standard suitable for continuous monitoring of respiratory rate that could easily be applied to the present study (Breteler et al., 2020; Marjanovic et al., 2020). Manual counting is used in hospital wards but is difficult to perform continuously and less accurate than the clinically acceptable limits (Lim et al., 2002; Kallioinen et al., 2021). Patient monitors based on impedance pneumography are commonly used in ICUs but are based on proprietary algorithms that introduce algorithm bias and data recording can be difficult. Capnography is regarded as the gold standard for measuring respiratory rate in intubated patients but is often difficult to use in non-intubated patients, as it is prone to frequent sensor malposition, high amounts of data loss, and poor patient acceptance (Breteler et al., 2020; Marjanovic et al., 2020). Similar studies commonly use respiratory inductance plethysmography to overcome these issues (Chan et al., 2022). Although respiratory inductance plethysmograph can be affected by sensor malposition, using belts on both the thorax and abdomen provides redundancy. Given that patients in the present study were free to move in bed, increasing the risk of data loss, we opted to use respiratory inductance plethysmograph belts in combination with nasal pressure flow for added reliability.

Nox T3s was chosen as a suitable device to extract reference respiratory rates. While Nox T3s is not approved for monitoring respiratory rate in hospital wards, it is medically approved for collecting respiration data during rest in bed and does not interfere with the standard of care. It is multichannel which enables recording of both respiratory inductance plethysmograph (thorax and abdomen) and nasal pressure flow with one device. Respiratory rates were calculated separately from each of the three raw signals in a multistep process designed to be like manual counting to ensure valid respiratory rates. First, outliers from movements were removed if they were outside the 1st or 99th percentile. Then, waveforms were centred around zero by applying a high-pass filter and smoothed with a Savitzky-Golay filter. Lastly, peaks were detected, and the respiratory rate was calculated by counting peaks over 1 minute. If an insufficient number of peaks were detected within a minute to estimate the respiratory rate, no respiratory rate value was supplied. Finally, a combined respiratory rate was calculated by taking the median of the respiratory rate from the three signals. A new respiratory rate was generated every second based on the last minute. Reference respiratory rates were calculated blinded from Guardian M10 respiratory rates. Respiratory rates from the reference were synchronised in time with Guardian M10 respiratory rates via a temporal cross-correlation of movements from Nox T3s (from built-in accelerometer) with movements calculated from raw radar data from Guardian M10. The reference respiratory rates were shifted in time by the time lag that maximised this cross-correlation.

### 2.5 Statistical analysis

A data analysis and statistical plan was written and submitted to the Norwegian Medical Products Agency and the regional ethical committee before the study started. The two device configurations (stationary and mobile) were analysed separately.

#### 2.5.1 Sample size

Agreement between the Guardian M10 and the reference was calculated using Bland-Altman 95% limits of agreement, as recommended for analysis of continuous variables (Bland and Altman, 1986; Ranganathan et al., 2017). The sample size was set to the smallest number of patients that would allow the study to show with a power of 0.8 (80% probability) that the limits of agreement with 95% confidence intervals (significance level of α = 0.05) were inside ±1.2 breaths min^−1^, in line with common recommendations (Bland and Altman, 1986; Gerke, 2020). While ± 3 breaths min^−1^ is typically used as a clinical acceptable limit, ±1.2 breaths min^−1^ was chosen as this should increase the sensitivity and specificity of detecting different clinical events.

A dataset owned by Vitalthings was used to calculate the required sample size. Since the data were not normally distributed (visually inspected with a histogram plot and a quantile-quantile plot), we used non-parametric bootstrapping as proposed for Bland-Altman analysis (Olofsen et al., 2015). First, 10,000 clinical trials were simulated per n (number of potential patients) by selecting n patients from the dataset with replacement. Then, for each simulated trial, the average number of measurements per patient were sampled with replacement from the data of each sampled patient (to account for different measuring time per patient). For each simulated trial, the bias was calculated as the mean difference and the limits of agreement were calculated using the 2.5 and 97.5 percentiles of the differences. The required number of patients was then the smallest number of patients (n) for which more than 80% of the simulated trials had agreements within the predefined limits, which gave a sample size of 36 patients. Further, we assumed that 10% of the patients would not be able to be measured for 30 min due to examinations and transfers from the emergency ward, meaning we needed to recruit 40 patients.

#### 2.5.2 Agreement analysis

To calculate the Bland-Altman limits of agreement and their 95% confidence intervals, a similar bootstrapping technique as above was used. First, 10,000 clinical trials were simulated by selecting *n* patients with replacement (*n* now fixed as the number of actual patients). Then, for each simulated trial, the actual average number of measurements per patient were sampled with replacement from the data of each sampled patient (to account for different measurement time per patient). Bias and limits of agreement were then calculated per simulated trial. Finally, the overall bias and limits of agreements were calculated by taking the mean of the trials (i.e., the upper limit of agreement was calculated as the mean of the 10,000 trials’ upper limits of agreements), and the 95% confidence interval of the limits were calculated by taking the 2.5 and 97.5 percentile of the trials’ respective limits of agreements (i.e., the lower 95% confidence interval of the upper limit of agreement was calculated as the 2.5 percentile of the 10,000 trials’ upper limits of agreement). Bland-Altman assumes independent measurements, and therefore measurements were sampled once every minute in the Bland-Altman plot, but all the measurements were used in the bootstrapping to avoid any sampling bias. A linear-mixed effect model with patient as random effect was considered, but not used, as the between-subject variance was negligible compared to the within-subject variance (intra-class correlation<0.05). Limits of agreement within ± 3 breaths min^−1^ were deemed clinically acceptable.

To simplify comparison with other studies and medical devices, agreement was also calculated as mean absolute error, mean relative absolute error (absolute error/reference value), root mean square error, and accuracy (% of clinically acceptable measurements, defined below).

#### 2.5.3 Clinical performance

In accordance with similar research, additional analyses were performed to validate clinical performance (Breteler et al., 2020; Chan et al., 2022). To evaluate potential clinical consequences, a Clarke Error Grid analysis was conducted (Clarke et al., 1987). The Clark Error Grid was adapted to respiratory rate monitoring by adjusting the zones according to the default alarm limits for low respiratory rate (bradypnoea <8 breaths min^−1^) and high respiratory rate (tachypnoea >30 breaths min^−1^) in the Guardian M10. The alarm limits are based on the standard early warning score systems (Physicians, 2021; García-Del-Valle et al., 2021), but with the thresholds slightly adjusted to reduce alarm fatigue (Burgess et al., 2009). The clinically acceptable limit was set to ±3 breaths min^−1^ or within 10% of the reference respiratory rate. The percentage of measurements in each zone was calculated. To analyse the concordance of trends, a four-quadrant plot was created for 1-minute trends with an exclusion zone of ± 1 breaths min^−1^ (Saugel et al., 2015). The overall concordance was calculated without the measurements in the exclusion zone. To evaluate the continuity of measurements, the gaps between valid measurements were calculated and grouped by duration (<5, 5–15, 15–60 min, 1–4 h). The Clark-Error and concordance analyses were performed on independent data sampled every minute, while the gap analysis was based on all the data.

## 3 Results

A total of 41 patients were recruited in the study, and 9 patients were excluded for being monitored for less than the predefined limit of 30 min. Clinical characteristics of the patients such as age, sex, body mass index, average respiratory rate, emergency ward admission diagnosis, and relevant past medical history are described in Table 1.

**TABLE 1:** Patient characteristics. Values are number (proportion) or mean ± SD (range).

Sex; male	16 (50%)
Age; year	52.4 ± 21.2 (18.0 to 85.0)
Height; cm	171.5 ± 9.8 (150.0 to 190.0)
Weight; kg	78.7 ± 18.3 (50.0 to 122.0)
BMI; kg m^−2^	26.6 ± 5.4 (18.4 to 43.7)
Average respiratory rate; breaths min^−1^	15.4 ± 3.8 (8.8 to 27.6)
Admission diagnosis	
Cardiac event	5 (16%)
Chest pain	8 (25%)
Abdominal pain	6 (19%)
Inflammation	2 (6%)
Syncope	1 (3%)
Other	10 (31%)
Past medical history	
Atrial fibrillation	5 (16%)
Obstructive sleep apnoea	1 (3%)
Asthma	5 (16%)
COPD	0 (0.0%)

BMI, body mass index, COPD, chronic obstructive pulmonary disease.

The patients were monitored for a median time of 42 min (IQR 35–46 min). The Guardian M10 had a median coverage (time with valid respiratory rate/valid monitoring time) of 89.8% (IQR 68.1%–95.7%) and 87% (IQR 79.8%–97.0%) for the stationary and mobile configuration, respectively. The reference had a median coverage of 98.9% (IQR 97.0%–99.7%), and a median of 0.3% and 0.4% of the Guardian M10 measurements were not validated for the stationary and mobile configuration, respectively. Full information on the data quantity is shown in Table 2.

**TABLE 2:** Data quantity. Values are median [IQR, range].

Total monitoring time; min	43.2 (38.3 to 54.5, 30.4 to 85.4)
Data removed due to deviations from protocol; min	0.2 (0.0 to 8.8, 0.0 to 33.6)
Valid monitoring time; min	41.6 (35.1 to 45.7, 30.2 to 59.2)
Coverage Guardian M10 stationary; %	89.8 (68.1 to 95.7, 26.6 to 99.1)
Coverage Guardian M10 mobile; %	87.0 (79.8 to 97.0, 34.0 to 99.3)
Coverage RIP thorax; %	94.7 (85.5 to 98.8, 0.0 to 99.7)
Coverage RIP abdomen; %	96.1 (84.0 to 98.7, 0.0 to 99.7)
Coverage RIP pressure flow; %	96.3 (88.3 to 98.9, 0.0 to 99.7)
Coverage reference; %	98.9 (97.0 to 99.7, 80.5 to 99.8)
Guardian M10 stationary measurements not validated; %	0.3 (0.2 to 0.5, 0.0 to 2.1)
Guardian M10 mobile measurements not validated; %	0.4 (0.2 to 0.7, 0.0 to 4.9)

Coverage, time with valid respiratory rate measurement divided by valid monitoring time, RIP, respiratory inductance plethysmography.

### 3.1 Agreement analysis

**FIGURE 2:**
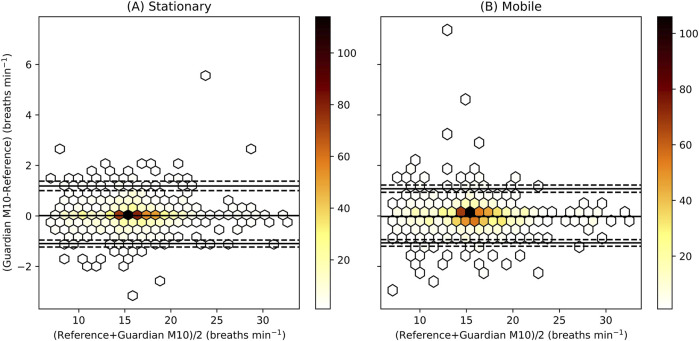
Bland-Altman plot for the stationary **(A)** and mobile **(B)** configuration. The colour illustrates the number of measurements inside each hexagon. The middle solid black line represents the bias, and the two other solid black lines the Bland-Altman limits of agreement with their 95% CI in dashed black lines. The limits of agreement are calculated using bootstrapping on all the data (*n_patients* = 32), while the hexagons represent only independent measurements (A, *n_measurements* = 1,112, B, *n_measurements* = 1,119) sampled every minute.

The bias was 0.1 breaths min^−1^ for the stationary (*n* = 1,112 measurements) and 0.0 breaths min^−1^ for the mobile (*n* = 1,119 measurements) configuration. Bland-Altman limits of agreement with 95% confidence interval ranged from −1.09 (−0.95 to −1.23) to 1.19 (1.00–1.38) breaths min^−1^ for the stationary configuration and from −1.07 (−0.93 to −1.20) to 1.07 (0.93–1.22) breaths min^−1^ for the mobile configuration. Figure 2 displays the Bland-Altman plots.

The mean absolute error was 0.31 breaths min^−1^, and the mean relative absolute error was 0.02 for both configurations. The root mean square error was 0.55 breaths min^−1^ and 0.52 breaths min^−1^ for the stationary and mobile configuration, respectively. The accuracy was 99.7% for the stationary configuration and 99.9% for the mobile configuration.

### 3.2 Clinical performance

**FIGURE 3:**
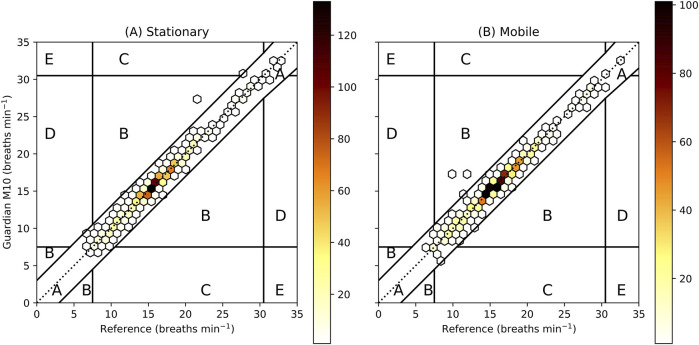
Clarke-Error Grid for the stationary (**A**, n_patients = 32, n_measurements = 1,112) and mobile (**B**, n_patients = 32, n_measurements = 1,119) configuration. The grid is adapted according to Guardian M10 alarm thresholds set to <8 for bradypnoea and >30 for tachypnoea. Zone A shows clinically acceptable measurements (within ±3 breaths min^−1^ or 10%), Zone B measurements that are clinically unacceptable but would not lead to false/missed/wrong alarm, Zone C false alarm, Zone D missed alarm and Zone E wrong alarm. The colour represents the number of measurements within each hexagon.

The Clark-Error grids (Figure 3) had 2 (0.2%) and 3 (0.3%) measurements in Zone B (clinically unacceptable measurements that would not lead to a false/missed/wrong alarm) for the stationary and mobile configuration, respectively. The remaining measurements were all in Zone A (clinically acceptable measurements within ±3 breaths min^−1^ or 10% of the reference).

**FIGURE 4:**
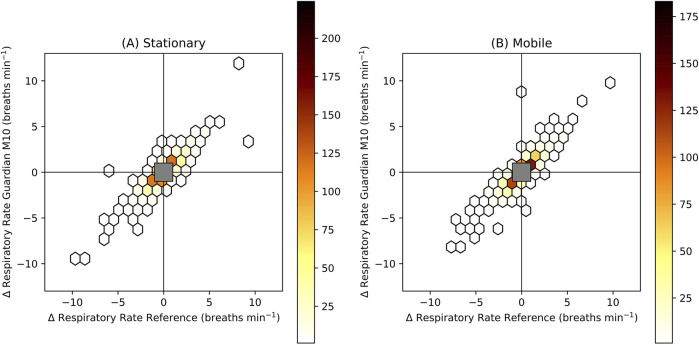
Four-quadrant plot for the stationary (**A**, *n_patients* = 32, *n_measurements* = 969) and mobile (**B**, *n_patients* = 32*, n_measurements* = 991) configuration. The values represent trends calculated as the change of respiratory rate over 1 min. The colour represents the number of measurements within each hexagon. An exclusion zone is added with limits ±1 breaths min^−1^. The concordance excluding values in the exclusion zone (measurements in the upper right quadrant and lower left quadrant/total measurements) was 95.5% and 95.7% for A and B, respectively.

For the trend analysis, the overall concordance was 95.5% and 95.7% for the stationary and mobile configuration, respectively. The four-quadrant plot is shown in Figure 4.

The gap analysis showed that there were no gaps between valid respiratory rate measurements longer than 5 min for either configuration.

No technical alarms were generated during the study.

## 4 Discussion

In this study, the Guardian M10 measured respiratory rate with a bias of 0.1 and 0.0 breaths min^−1^ and 95% limits of agreement ranging from −1.1 to 1.2 and −1.1 to 1.1 breaths min^−1^ for the stationary (*n* = 1,112 measurements) and mobile configuration (*n* = 1,119 measurements), respectively. No false or technical alarms were generated, and no alarms were missed. Trends showed high concordance, and no gap between valid measurements was longer than 5 min.

The limits of agreement were significantly narrower than the inter-observer differences shown for the standard of care (manual counting) with limits of agreement ranging from −4.2 to 4.4 breaths min^−1^ (Lim et al., 2002; Kallioinen et al., 2021). The limits of agreement were also narrower than for all 31 wearable devices and for all 13 contactless devices included in a recent review, where most devices had limits of agreements outside the acceptable limits of ±3 breaths min^−1^ (Chan et al., 2022). In the review, two of the contactless devices seemed to have similar agreement to the present study, but one did not provide limits of agreement (Zhu et al., 2019), and the other investigated nightly averages, not instantaneous values (Khushaba et al., 2017). A study found limits of agreement as narrow as −0.07 to −0.04 breaths min^−1^ for nightly averages, compared with −0.94 to 0.80 breaths min^−1^ for instantaneous measurements during sleep (Toften et al., 2022).

The study concluded with 32 patients, four fewer than the initial estimate required to achieve 95% confidence intervals within ±1.2 breaths min^−1^ for the limits of agreement. Although we considered recruiting more patients, the upper limit for the stationary configuration at 1.19 breaths min^−1^ made it unlikely that additional patients would narrow the interval sufficiently, so the study was concluded. For all other purposes, the sample size should be more than sufficient. The narrow 95% confidence intervals leave little uncertainty for most clinical applications. The 32 patients provided 1,112 independent measurements (no time overlap and negligible between-subject variance) for the stationary configuration and 1,119 for the mobile configuration. The patient group was diverse and representative of the general population, equally representing both sexes and covering a wide range of age, height, weight, body mass index, and medical conditions. The sample size is in line with previous studies, where a recent review on 56 studies on wearables and 29 studies on contactless devices had an average of 35 and 29 patients, respectively (Chan et al., 2022).

The respiratory rates ranged from 6 to 33 breaths min^−1^. This covers all the clinical zones of widely established early warning systems (Physicians, 2021; García-Del-Valle et al., 2021). Though, the study covered few values of bradypnoea (<8 breaths min^−1^) and tachypnoea (>30 breaths min^−1^) and did not cover the whole range of the Guardian M10 (2–60 breaths min^−1^). Though, to our knowledge, nothing indicates that the agreement would vary with the respiratory rate, which was also true in the present study. As the device had previously been validated on the whole range (2–60 breaths min^−1^) on a breathing robot (SimMan with ASL 5000 Lung Solution, Laerdal Medical, Norway), covering the whole range was not the focus of this study. Collecting more instances of bradypnoea and tachypnoea can be challenging when validating medical equipment, as in the present study, as the device would have to alert nurses when the thresholds for bradypnoea or tachypnoea are breached. The nurses would then start necessary treatment to prevent further deterioration, possibly preventing sustained low or high respiratory rates.

Although the Guardian M10 displays respiratory waveforms that could detect patterns like Cheyne-Stokes, analysing these patterns was beyond the scope of this study and subject to future research.

Contactless monitoring of respiratory rate has several potential advantages. First, the device should be tolerable for all types of patients (non-discriminating) as nothing is attached to the body, including patients with dementia, delirium, burns, and skin allergies. Second, there is no need for direct contact between the nurse and the patient, reducing the risk of transmitting diseases or infections to or from patients. Third, the Guardian M10 contains no single-use components disposed of after use (including batteries) and no chemical disinfection is needed between patients, potentially reducing the environmental impact. On the other hand, the device cannot monitor patients if they leave the monitoring area. However, patients typically sit or lie down 85%–100% of the time (Fazio et al., 2020). Moreover, previous research indicates that monitoring during out-of-bed activities triggers clinically irrelevant alarms (Drew et al., 2014).

Respiratory inductance plethysmography and pressure flow was deemed the best option as a reference device, as rationalized in the section 2.4 Reference. While similar studies using respiratory inductance plethysmography did not use pressure flow (Chan et al., 2022), pressure flow increased the coverage in the present study. If the reference device has low coverage, there is a risk of not being able to validate many of the measurements, especially during challenging measurement periods, potentially skewing results to appear more favourable. In our study, the agreement of the device varied from ±0.1 breaths min^−1^ (bias) to ±1.2 breaths min^−1^ (Bland-Altman 95% limits of agreement) depending on how agreement was defined. Transparency and openness from manufacturers on how the agreement of their device was calculated and what dataset it was derived from would enable clinicians to make more informed decisions on which equipment to use in which situations. The manufacturer-specified agreement can differ significantly from the agreements found in independent studies (Breteler et al., 2020).

The present study demonstrated that Guardian M10 can monitor respiratory rates accurately and continuously in a hospital setting. While research shows that there is a significant clinical benefit of continuous monitoring of respiratory rate, follow up studies across multiple hospitals and wards should be conducted to quantify the Guardian M10’s clinical impact in terms of increased patient safety, nurse workload, environmental aspects and reduced hospital cost.

## Data Availability

The raw data supporting the conclusions of this article will be made available by the authors, without undue reservation.
